# Peptide Triazole Thiol Irreversibly Inactivates Metastable HIV-1 Env by Accessing Conformational Triggers Intrinsic to Virus–Cell Entry

**DOI:** 10.3390/microorganisms9061286

**Published:** 2021-06-12

**Authors:** Charles Gotuaco Ang, Erik Carter, Ann Haftl, Shiyu Zhang, Adel A. Rashad, Michele Kutzler, Cameron F. Abrams, Irwin M. Chaiken

**Affiliations:** 1 Department of Biochemistry and Molecular Biology, College of Medicine, Drexel University, Philadelphia, PA 19102, USA; ec899@drexel.edu (E.C.); arh97@drexel.edu (A.H.); sz399@drexel.edu (S.Z.); adel.rashad917@gmail.com (A.A.R.); 2 School of Biomedical Engineering, Science, and Health Systems, Drexel University, Philadelphia, PA 19102, USA; 3 Departments of Medicine and Microbiology and Immunology, College of Medicine, Drexel University, Philadelphia, PA 19102, USA; mak98@drexel.edu; 4 Department of Chemistry, College of Arts and Sciences, Drexel University, Philadelphia, PA 19102, USA; 5 Department of Chemical and Biological Engineering, College of Engineering, Drexel University, Philadelphia, PA 19102, USA; cfa22@drexel.edu

**Keywords:** HIV-1, Env, metastability, flow cytometry, entry inhibition, membrane poration, peptide triazole, MPER, 6-helix bundle

## Abstract

KR13, a peptide triazole thiol previously established to inhibit HIV-1 infection and cause virus lysis, was evaluated by flow cytometry against JRFL Env-presenting cells to characterize induced Env and membrane transformations leading to irreversible inactivation. Transiently transfected HEK293T cells were preloaded with calcein dye, treated with KR13 or its thiol-blocked analogue KR13b, fixed, and stained for gp120 (35O22), MPER (10E8), 6-helix-bundle (NC-1), immunodominant loop (50-69), and fusion peptide (VRC34.01). KR13 induced dose-dependent transformations of Env and membrane characterized by transient poration, MPER exposure, and 6-helix-bundle formation (analogous to native fusion events), but also reduced immunodominant loop and fusion peptide exposure. Using a fusion peptide mutant (V504E), we found that KR13 transformation does not require functional fusion peptide for poration. In contrast, simultaneous treatment with fusion inhibitor T20 alongside KR13 prevented membrane poration and MPER exposure, showing that these events require 6-helix-bundle formation. Based on these results, we formulated a model for PTT-induced Env transformation portraying how, in the absence of CD4/co-receptor signaling, PTT may provide alternate means of perturbing the metastable Env-membrane complex, and inducing fusion-like transformation. In turn, the results show that such transformations are intrinsic to Env and can be diverted for irreversible inactivation of the protein complex.

## 1. Introduction

While there have been admirable strides in understanding HIV-1′s replication mechanisms and advances in virus suppression, and infection control, HIV-1 has persisted globally, with 2 million new infections per year [1,2]. The most effective tool so far in controlling HIV-1 infection has been combination antiretroviral therapy (cART), a drug cocktail targeting multiple steps of the viral infection cycle. However, a limitation of cART is that all of the inhibitor components of reverse transcriptase or integrase act after viral entry into the target cell and must be in the target cell simultaneously with the viral RNA. Entry inhibitors, a developing class of anti-HIV treatments, are intended to intervene earlier, targeting virus directly at the externally presented viral Env protein complex before cell entry [3,4].

Env is the “signature” surface protein of HIV-1, being both the mediator of entry into target CD4-positive cells, as well as the only surface-expressed protein from the viral genome. Conformationally, Env “spikes” are composed of six parts, a trimer of dimers. Each Env dimer is composed of gp120 and gp41, cleaved and processed from a single parent protein, gp160. When assembled, the Env trimer spike is in a metastable state [5,6,7,8,9], whereby perturbation by a series of interactions with CD4 and co-receptor on a target cells will induce gp120 shedding, gp41 conformational rearrangement, and energy release necessary to open a fusion pore between virus and cell, gaining entry for the viral RNA and reverse transcriptase [10]. As such, targeting and inactivating the Env protein complex could provide an important means to control HIV infection and progression. Most entry inhibition strategies have focused primarily on targeting gp120′s CD4 and/or co-receptor binding sites [11,12,13,14,15], or interrupting 6-helix-bundle formation by gp41 [16,17,18]. Some efforts have also been aimed at targeting the highly conserved membrane proximal external region (MPER) of gp41, which is at the base of the gp41 ectodomain, partially buried in the membrane, and is the binding site of several broadly neutralizing antibodies against HIV-1 [19,20,21]. In contrast, we have investigated the potential to trigger conformational rearrangements and inactivating responses based on the known metastability of the Env protein complex.

**Figure 1:**
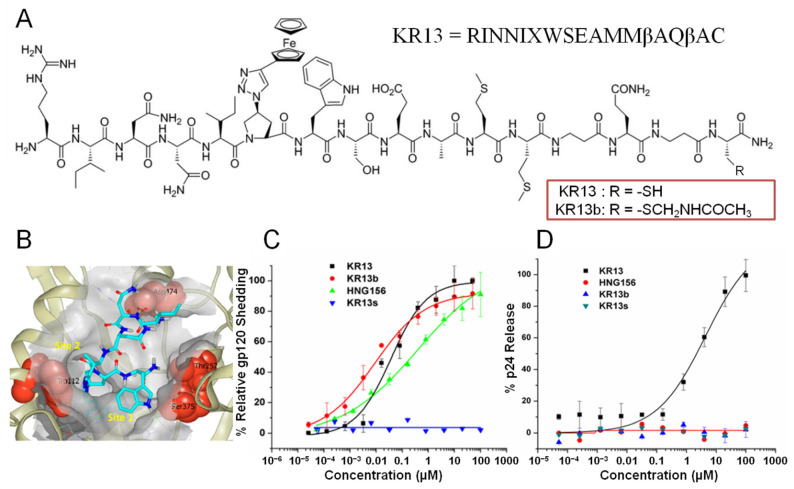
(**A**) Chemical structure and amino acid sequence of peptide triazole thiol (KR13) and blocked peptide triazole thiol (KR13b) compounds [22,23]. Reprinted (adapted) with permission from Bastian, A.R.; Kantharaju; McFadden, K.; Duffy, C.; Rajagopal, S.; Contarino, M.R.; Papazoglou, E.; Chaiken, I. Cell-free HIV-1 virucidal action by modified peptide triazole inhibitors of Env gp120. ChemMedChem 2011, 6, 1335-1339, 1318, doi:10.1002/cmdc.201100177. Copyright 2011 Wiley-VCH Verlag GmbH& Co. KGaA, Weinheim. (**B**) Localization of the IXW pharmacophore of a minimized peptide triazole to gp120′s Phe43 pocket [27]. Hot spots Trp112, Thr257, and Asp474 are shown as CPK in red. The Trp residue rests in site 1, whereas the triazole moiety is buried in site 2. Reprinted (adapted) with permission from Aneja, R.; Rashad, A.A.; Li, H.; Kalyana Sundaram, R.V.; Duffy, C.; Bailey, L.D.; Chaiken, I. Peptide Triazole Inactivators of HIV-1 Utilize a Conserved Two-Cavity Binding Site at the Junction of the Inner and Outer Domains of Env gp120. Journal of medicinal chemistry 2015, 58, 3843–3858, doi:10.1021/acs.jmedchem.5b00073. Copyright 2015 American Chemical Society. (**C**) Env gp120 shedding and (**D**) Virolysis of BaL.01 pseudovirus by KR13, non-thiol parent PT HNG156, blocked-thiol PTT KR13b, and IWX scrambled pharmacophore PTT KR13s [23]. Figures in C and D reproduced with permission from Bastian, et al., Retrovirology; published by BioMed Central, Ltd. 2013 (CC-BY).

KR13 (Figure 1A) is a peptide triazole thiol (PTT) entry inhibitor that has been shown to cause irreversible inactivation of HIV-1 by two methods, gp120 shedding and virus lysis [22,23]. PTT compounds and their parent group of peptide triazole (PT) compounds feature the IXW pharmacophore (X = ferrocenyl-triazole-Pro), which occupies subsites in the CD4 binding site interface of HIV-1 Env (Figure 1B), disrupts the co-receptor binding site allosterically, and induces gp120 shedding (Figure 1C), irreversibly inactivating the virus [24,25]. PTTs, including KR13, are distinguished from PTs by possessing a terminal free thiol. Strikingly, PTT compounds were discovered to cause lytic breakdown of treated viruses, releasing their contents under cell-free conditions (Figure 1D) and suggesting cascading interactions with Env’s native disulfides [22,23,26]. To facilitate mechanistic studies with KR13, the variant KR13b was synthesized with the acetamidomethyl protecting group still attached to the cysteine thiol (Figure 1A) to prevent the possibility of disulfide exchange. Prior studies have shown KR13b to function identically to other non-thiol PT compounds (i.e., KR13b inhibits infection by HIV-1, causes gp120 shedding, but does not cause virus lysis/p24 leakage), and it was considered as such in this study [23]. In order to understand these desirable effects of irreversible virus inactivation, several investigations into PT and PTT mechanisms have already been performed and lay the groundwork for the current study. In particular, two notable findings have suggested that the PTT transformation may be tapping into components of the same native mechanisms that Env uses for fusion and entry. First, KR13-induced lysis is inhibited by the addition of 6-helix-bundle formation inhibitor T20 [23]; and second, mutational studies have identified the disulfide pairs of C296-C331 and C598-C604 as being required for the lytic function of KR13. Both of the latter also are important for function in the native fusion process [26]. From the above, we formed the hypothesis that the conformational transformations induced by PTT compounds access intrinsic properties of the metastable Env to trigger its inactivation.

Experiments with PT and PTT compounds have thus far primarily been focused on their role as virus entry inhibitors; however, due to their targeting of the surface-exposed protein Env, we have been expanding the scope of experiments to include Env-presenting cells as potential tools and platforms for mechanistic investigation, vaccine development, and cell sensitization. Mechanistically, in this study, we sought to validate past studies on PT and PTT, and track epitopes in key Env regions and membrane intactness, and in doing so learn the extent to which PTT-induced transformation might echo and involve the native fusion transformations of Env. Thorough understanding of the post-transformation Env conformation could then open options for PT and PTT studies on producing immunogenic Env for presentation (e.g., on cells, virus, or nanodisc carriers), or as a potential “kill” treatment or supplement, by disrupting the membrane and exposing vulnerable epitopes.

## 2. Materials and Methods

### 2.1. Production and Validation of KR13 and KR13b

KR13 and KR13b peptides were synthesized with a Liberty Blue microwave peptide synthesizer (CEM; Matthews, NC, USA), using standard Fmoc chemistry on Rink amide resin (Chem-Impex; Wood Dale, IL, USA), and purified to >95% homogeneity by analytical reverse-phase HPLC on a C18 column (Waters Corporation; Milford, MA, USA) [22,23,26].

### 2.2. Cell Preparation

The plasmid encoding WT JRFL Env was a kind gift from Dr. Joseph Sodroski. HEK293T cells (CRL-3216, human embryonic kidney 293 cells with SV40 T-antigen expression) were obtained from ATCC (Manassas, VA, USA). Polyethylenimine (PEI) 25kDa linear polymer was obtained from Polysciences, Inc. (Warrington, PA, USA). All other reagents, unless otherwise noted, were obtained from Fisher Scientific (Hampton, NH, USA).

HEK293T cells were seeded at 3 million cells per T75 flask, transiently transfected with 4 µg of JRFL Env plasmid DNA and 48 µL PEI (1 mg/mL solution), adapted from the pseudovirus production protocol used in prior studies [22,23,26,28]. Cells were detached with 5 mM EDTA in DPBS at 24 h after transfection and reseeded at 50,000 cells per well in 48-well plates. Transfection efficiency was measured at 90–95% by flow cytometry.

### 2.3. Flow Cytometry Assays

Transfected HEK293T cells were incubated with 1 µM calcein acetoxymethyl-ester (Calcein AM; Thermo Fisher Scientific; Waltham, MA, USA) for 30 min to load cells with fluorescent dye, washed with media, and incubated for 4 h at 37 °C with serial dilutions of KR13, KR13b, or PBS control. After incubation, cells were washed with media and allowed to rest for 1 h at 37 °C in media after treatment so any in-progress transformations could finish. After resting, cells were detached with 5mM EDTA, washed and resuspended in flow cytometry buffer (1% BSA and 1mM EDTA in 10mM PBS, pH 7.4), fixed with 2% fresh paraformaldehyde in PBS (15 min at room temperature), and then washed and resuspended three times. Fixed cells were then stained with selected antibodies (35O22, 10E8, 50-69, NC-1, sourced from NIH AIDS Reagent Program, Manassas, VA, USA; VRC34.01 was a kind gift from Dr. Peter Kwong, Vaccine Research Center, NIAID/NIH, Bethesda, MD, USA; isotype control for human IgG1 from Novus Biologicals, Littleton, CO, USA; isotype control for mouse IgG2α from R&D Systems, Minneapolis, MN, USA) for 1 h at room temperature and 5 µg/mL, followed by three washes in flow cytometry buffer, then incubation with secondary stains anti-Hu IgG1 Alexa 488, anti-Ms IgG2 Alexa 488, or anti-Hu IgG1 PerCP (Jackson ImmunoResearch Laboratories, Inc.; West Grove, PA, USA) for 1 h, at room temperature, and 1:500 dilution. Cells were washed a final three times and resuspended in flow cytometry buffer before measuring retained calcein fluorescence and antibody signal in counted cells on a Guava EasyCyte 5HT flow cytometry system (Millipore; Burlington, MA, USA). Cells analyzed were subject to forward/side scatter gating based on untreated control populations of identically transfected cells, and median fluorescence values were obtained by using Guava InCyte 3.2 software.

### 2.4. Virus Production for V504E Mutants

The plasmid encoding the NL4-3.LucAM.R^−^E^−^ backbone was obtained from the NIH AIDS Reagent Program (Manassas, VA, USA). The V504E mutant in JRFL Env (V513E by HXBC2 standard numbering) was made by site-directed mutagenesis, using the aforementioned JRFL Env plasmid and the forward primer (GTG to GAG) and reverse primer (CAC to CTC), sourced from Integrated DNA Technologies, Inc. (Coralville, IA, USA):

Forward: 5′-GA GAA AAA AGA GCA G**A**G GGA ATA GG-3′

Reverse: 3′-CT CTT TTT TCT CGT C**T**C CCT TAT CC-5′

Presence of the mutation in the resulting plasmid was confirmed with sequencing by GENEWIZ (South Plainfield, NJ, USA).

HEK293T cells were seeded at 3 million cells per flask, transiently transfected with 4 µg of JRFL WT Env or JRFL V504E Env plasmid DNA, 8 µg of backbone plasmid DNA, and 48 µL PEI (1 mg/mL solution), per the pseudovirus production protocol used in prior studies [22,23,26,28]. Supernatants were collected 48 h after transfection and purified by filtration on a 100 kDa concentrator, then by a 6–20% iodixanol gradient spun at 30,000 RPM for 2 h using an Optima L-100K ultracentrifuge with SW41 Ti swinging-bucket rotor (Beckman-Coulter; Indianapolis, IN, USA). The fractions containing 13% to 18.6% iodixanol were pooled, frozen, and quantified for p24 content as the produced pseudovirus.

### 2.5. ELISA Detection of p24 for Quantification of Virolysis

ELISA plates were obtained from Corning, Inc. (Corning, NY, USA). Anti-p24 mouse and rabbit IgG antibodies were obtained from Abcam (Cambridge, UK). Anti-rabbit HRP-linked secondary antibodies were obtained from GE Life Sciences (Marlborough, MA, USA). The 1,2-phenylenediamine was obtained from Sigma-Aldrich (St. Louis, MO, USA). All other reagents, unless otherwise noted, were obtained from Fisher Scientific (Hampton, NH, USA).

ELISA plates (96-well) were prepared by coating with 50 ng per well of anti-p24 mouse antibody (suspended in PBS) for 18 h at 4 °C, then blocked by incubating in 3% BSA in PBS for 2 h, at room temperature. Virus samples (quantified at 500 ng of p24) were treated with KR13 or KR13b for 2 h at 37 °C. Samples were then pelleted at 21,300× *g* for 2 h at 4 °C with an Eppendorf 5425 R benchtop centrifuge (Enfield, CT, USA). The top 150 µL of supernatant was then removed from each pelleted sample, combined with 75 µL of 1% Triton-X, and then dispensed at 60 µL per well into prepared ELISA plates containing 20 µL of 3% BSA in PBS per well.

Samples were allowed to bind to the ELISA plate for 18 h at 4 °C, before removal of excess supernatant and addition of anti-p24 rabbit antibody, diluted 1:3000 in 0.5% BSA in PBS, and allowed to shake for 1 h at room temperature. Plates were washed 3 times with PBS, before addition of anti-rabbit HRP-linked IgG, diluted 1:3000 in 0.5% BSA in PBS, and allowed to shake for 1 h at room temperature. Plates were washed an additional 3 times before removal of any excess liquid and addition of 180 µL per well of 1,2-phenylenediamine dissolved in phosphate–citrate buffer with sodium perborate (0.4 mg/mL). Plates were covered with foil and allowed to shake for 30 min at room temperature, before reading colorimetric output on a Tecan Infinite F50 plate reader (Männedorf, Switzerland).

### 2.6. Data Plotting and Statistical Analysis

Data for dose-dependence curves were plotted by using OriginPro 8 (OriginLab; Northampton, MA, USA), where sufficient data (three or more independent experiments) were available, and fit to a logistic dose-dependence curve, using the Levenberg–Marquardt algorithm for nonlinear least-squares regression [29]. The logistic function fitted follows the below equation, where *A*_1_ and *A*_2_ are initial and final values, respectively, *x*_0_ is the value that yields the midpoint of *y*, and *p* is a growth or decay parameter describing the steepness of the curve:

(1) $$y=\frac{A_{1}-A_{2}}{1+{\left(x/x_{0}\right)}^{p}}+A_{2}$$

Calculations of EC_50_ are presented as *x*_0_ ± the standard error of *x*_0_.

Error bars on dose-dependence curves showing averages of multiple independent experiments depict the combined (pooled) sample standard deviation from each independent experiment, calculated as follows:

(2) $$s_{pooled}=\sqrt{\frac{s_{1}^{2}+s_{2}^{2}+\ldots +s_{n}^{2}}{n-1}}$$

## 3. Results

### 3.1. PTT KR13 Treatment Causes Transformation of Cell-Presented Env, Resulting in Membrane Disruption, Calcein Leakage, and Specific Epitope Exposure Changes in Env

Env-presenting cells were produced by modifying the Montefiori pseudovirus production protocol [28] to omit the NL4-3 backbone plasmid; HEK293T cells were transfected with only the Env-encoding plasmid. The known synthesis and trafficking pathways of Env account for no further processing or modification to the Env trimer between transport to cell surface and budding and assembly into free virions, and should leave Env in the same conformation [30,31]. While the cell membrane as a whole differs from the subset of regions that bud to form virus particles [32], there is evidence for the association of Env’s cytoplasmic tail with cholesterol even without supporting viral proteins such as Gag [31,33], and the functionality of cell–cell fusion assays as alternative or complement to virus–cell assays indicates a sufficiently similar Env–membrane complex that retains fusion functionality [34,35,36].

Much like our previous study [37], we sought to evaluate proven viral inhibitors that cause membrane disruption against Env-presenting virus in the context of Env-presenting cells. To address this possibility, we utilized a cellular dye leakage assay, using Calcein AM, which diffuses across cell membranes, and is then cleaved by intracellular esterases into a membrane impermeable and highly fluorescent form [38]. Transfected and non-transfected cells were pre-incubated for 30 min with 1 µM Calcein AM for cells to uptake and cleave the dye. Cells were then incubated with indicated concentrations of KR13 or KR13b for 4 h at 37 °C, before washing, plate detachment, and fixation with 2% paraformaldehyde. Cells were stained with monoclonal antibodies (5 µg/mL) reflecting different regions and conformations of the Env gp41 in order to gain insight into the post-treatment configuration of Env. Secondary staining was performed with anti-human IgG1-Alexa 488, anti-mouse IgG1-Alexa 488, or anti-human IgG-PerCP, as appropriate before analysis by flow cytometry. Transfection efficiency for these cells was 90–95%, as measured by flow cytometry.

Antibody (Ab) 35O22 was selected for its binding to the gp120/gp41 interface and used as an indicator for the presence of mature, intact Env spike [39,40]. Ab 10E8 was selected as a broadly neutralizing antibody that targets the gp41 MPER, and as one of the preferred epitopes for MPER-based vaccine development, due to its greater potency and breadth compared to the other broadly neutralizing MPER antibodies 2F5 and 4E10 [41,42]. Ab NC-1 was selected as an indicator of 6-helix-bundle formation, a key step in the native fusion process [43,44]; NC-1 is also very well characterized in comparison to other 6-helix-bundle-targeting antibodies currently available. Ab 50-69 was selected as an antibody indicative of the immunodominant loop (590-613) and specifically as one that recognizes conformations stabilized by the C598-C604 disulfide bond [44,45,46,47], previously established as a requirement of PTT-induced virus lysis [26]. Ab VRC34.01 was selected for its binding to the fusion peptide, the hydrophobic N-terminal of gp41 that inserts and anchors into the target cell’s membrane during native fusion [48,49,50].

**Figure 2:**
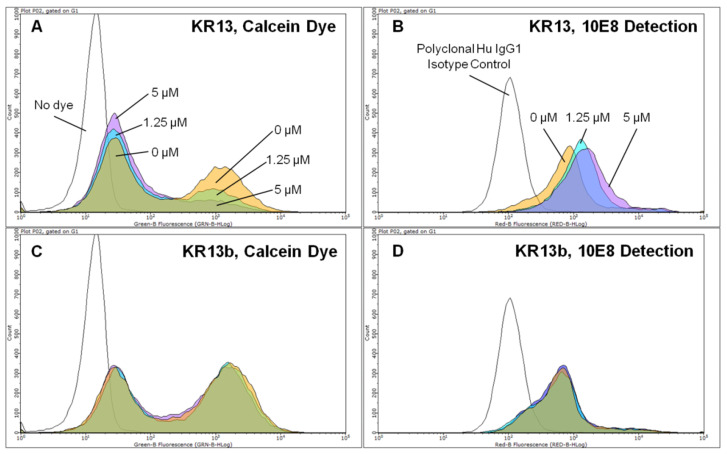
Sample histograms illustrating dose response to KR13 or KR13b at four concentrations. Open: No dye loaded/polyclonal Hu IgG1 isotype control. Orange: 0 µM inhibitor treatment. Cyan: 1.25 µM inhibitor treatment. Purple: 5 µM inhibitor treatment. (**A**) KR13 treatment, measuring for calcein retention. (**B**) KR13 treatment, measuring for 10E8 detection. (**C**) KR13b treatment, measuring for calcein retention. (**D**) KR13b treatment, measuring for 10E8 detection. Data shown are representative for one of four independent experiments. At least 5000 cells were examined for each condition in an independent experiment.

Figure 2 shows representative histograms obtained upon treatment with varying concentrations of either KR13 or KR13b, in addition to either non-dye loaded cells or polyclonal isotype control stained cells. As shown, untreated cells typically have a bidisperse distribution relative to retained calcein or staining. In Figure 2A, treatment with KR13 results in progressive loss of the high-calcein population, counterbalanced by growth of the low-calcein population. This can be directly contrasted with Figure 2C, wherein equivalent concentrations of KR13b treatment result in no change to the high- or low-calcein populations. Figure 2B shows the epitope exposure response of 10E8 to KR13 treatment: although beginning as a large low-10E8 population and a small high-10E8 population, treatment causes rightwards shift of the low-10E8 population and some increase in the high-10E8 population. Again, the contrasting effect of KR13b treatment is shown in Figure 2D, where there is neither shift nor change in the cell populations.

**Figure 3:**
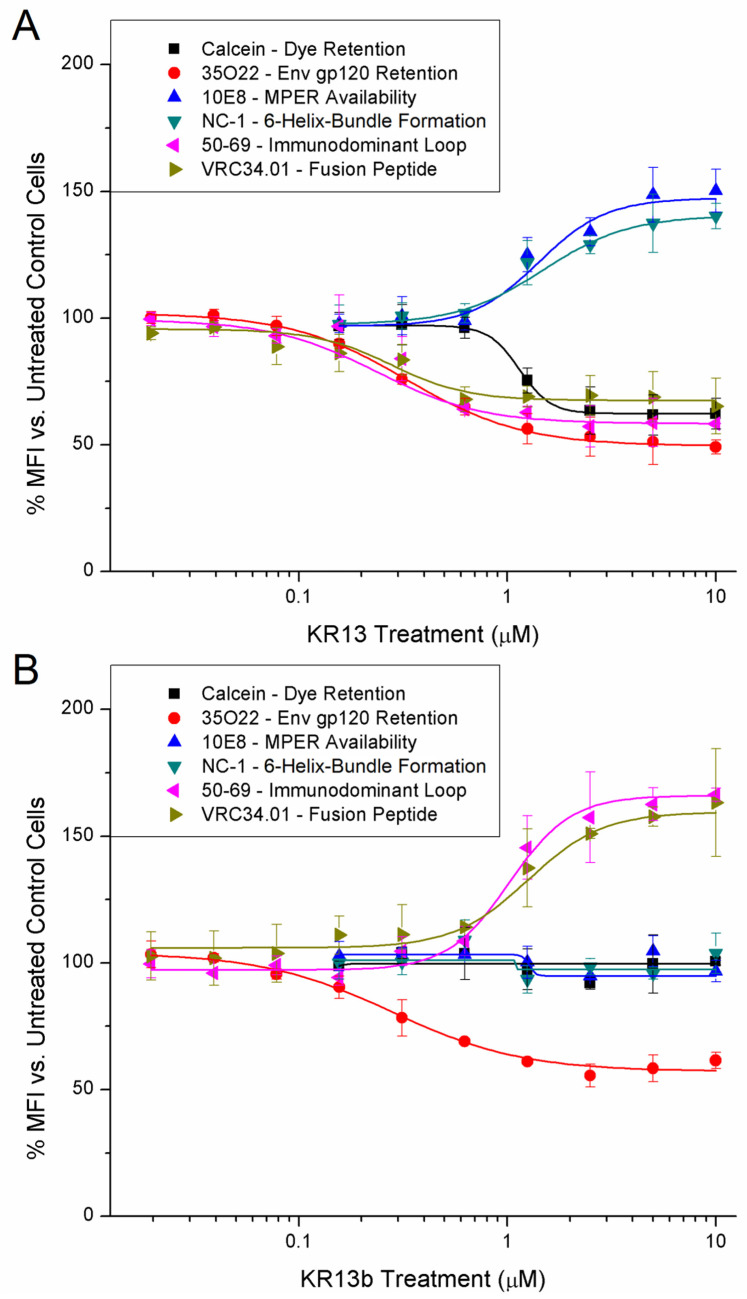
Treatment with KR13 (**A**) and KR13b (**B**) result in dose-dependent changes in epitope exposure and membrane disruption (for KR13). Cells were treated with compounds for 4 h at 37 °C before wash, retrieval, and fixation. Data shown are the average of four independent experiments, and error bars represent the standard deviation of the mean. At least 5000 cells were examined for each condition of an independent experiment.

Data from all selected antibodies were collated and graphed in Figure 3, showing comparative dose response in each antibody between equivalent KR13 and KR13b treatments (for individual graphs, see Supplementary Materials Figure S1). Tracking-retained intracellular calcein confirmed that the membrane disruption event seen in virus lysis [22] is transferable into cells presenting surface Env; however, unlike the effects of KR13 against viruses, no appreciable destruction of cells or cytotoxicity was observed with KR13 or KR13b treatment of Env-presenting cells, as determined by WST-1 mitochondrial activity assay (Takara Bio USA, Inc.; Mountain View, CA, USA) and flow cytometry cell counts (see Supplementary Materials Figure S2). Ab 35O22 showed roughly equal reduction in signal between KR13 and KR13b treatment, which agrees with the prior studies on peptide triazole compounds identifying gp120 shedding as the key observable effect of peptide triazole treatment and the effect responsible for infection inhibition [24,51,52]. 

As summarized in Table 1, KR13 treatment also increased 10E8 and NC-1 binding, but decreased 50-69 and VRC34.01 binding; KR13b treatment caused no apparent exposure change in 10E8 or NC-1 binding, but notably increased 50-69 and VRC34.01 binding. This would suggest that KR13 induces 6-helix-bundle formation and MPER exposure in Env, but also occludes the immunodominant loop and the fusion peptide. In contrast, KR13b appears to expose or stabilize the immunodominant loop and fusion peptide regions. This is particularly striking in light of fusion peptide’s role of cell membrane insertion during native infection; KR13 has been characterized as causing virus lysis in the absence of target cells, meaning fusion peptide would lack an opposing membrane [22].

### 3.2. Fusion Peptide Can Be Functionally Disabled while Retaining KR13-Induced Virolysis

**Figure 4:**
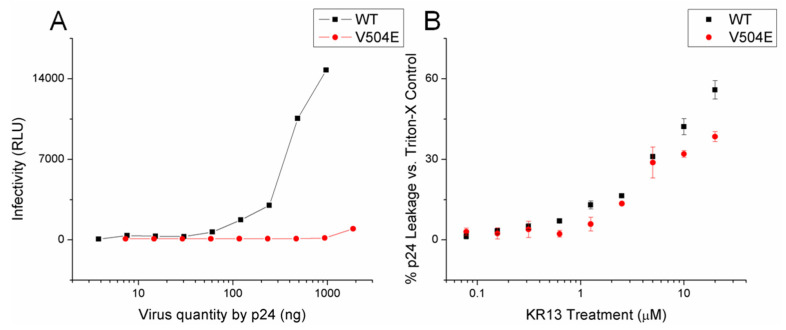
V504E JRFL fusion peptide nullification mutant pseudovirus loses infectivity but retains susceptibility to KR13. (**A**) Infectivity of produced pseudovirus. (**B**) Lytic response to KR13 treatment (2 h incubation, 500 ng p24 virus quantity). Data shown are the average of three independent experiments, and error bars represent the standard deviation of the mean.

To better understand fusion peptide’s role during KR13-induced conformational change, we produced the V504E mutant in JRFL Env by site-directed mutagenesis. V504E (V513E by HXBC2 numbering) has been shown to neutralize infection capability by rendering the fusion peptide too hydrophilic for membrane insertion [53,54]. As expected, V504E pseudovirus showed minimal infectivity compared to WT JRFL pseudovirus (Figure 4A); however, V504E pseudovirus remained susceptible to KR13-induced lysis, showing p24 leakage comparable to the WT control (Figure 4B). Our understanding of this result suggests that the KR13-induced conformational change does not require insertion of the fusion peptide into any membrane, either the Env-bearing cell’s own or that of an (absent) opposing membrane.

### 3.3. MPER Exposure and Membrane Disruption Occur Downstream of 6-Helix-Bundle Formation

**Figure 5:**
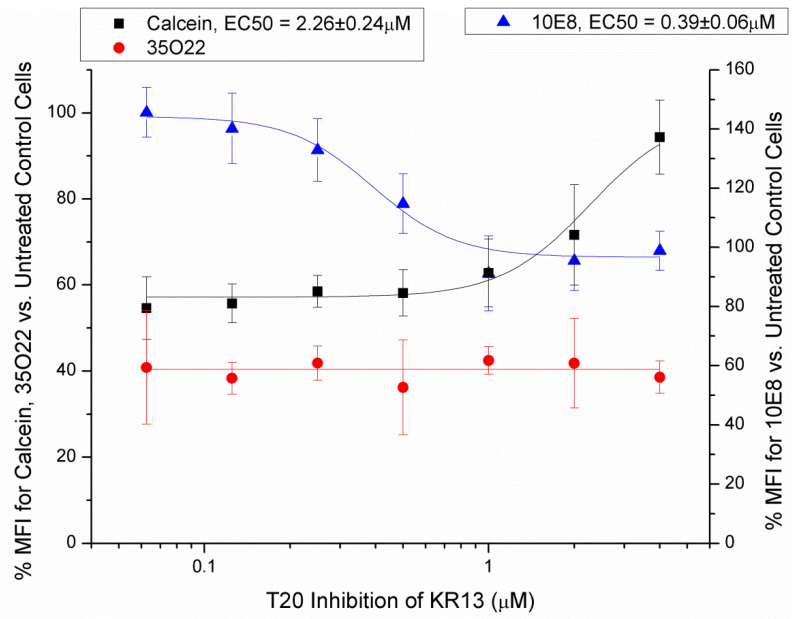
T20 inhibits gp41 and membrane transformations during KR13 treatment of Env-presenting cells. (Left *y*-axis) Flow cytometry detection of calcein retention (membrane integrity) and 35O22 (gp120 shedding). (Right *y*-axis) Flow cytometry detection of 10E8 (MPER). Data shown are the average of three independent experiments, and error bars represent the standard deviation of the mean. EC_50_ values are presented as *x*_0_ ± the standard error of *x*_0_.

Based on the notable effects of KR13 in eliciting membrane disruption, 6-helix-bundle formation, and MPER exposure, we treated JRFL-Env-presenting cells simultaneously with a static concentration of KR13 (4 µM) and serial dilutions of the 6-helix-bundle/fusion inhibitor T20 (top concentration of 4 µM). T20 itself is a derivative of the HR2 segment of gp41. It inhibits 6-helix-bundle formation by binding into the grooves of the exposed HR1 trimer and prevents native HR2 units from folding into said grooves. As shown in Figure 5, T20 inhibits KR13-induced membrane disruption (as measured by loss of calcein retention) and MPER exposure (as measured by 10E8), but not gp120 shedding (as measured by 35O22), suggesting that the former two events require and occur after 6-helix-bundle formation. This is in agreement with the current overall understanding of PTT-induced transformations as causing sequential effects of gp120 shedding (first form of irreversible inactivation), followed by further gp41 transformation and membrane poration (second form of irreversible inactivation).

## 4. Discussion

### 4.1. Env-Transfected Cells Function as Suitable Models for Evaluating Transformations of Surface Env and Membrane

Env-presenting cells provided a useful platform for the investigation of surface Env trimer and membrane transformations, remaining intact after inhibitor treatment and facilitating robust epitope detection via flow cytometry. This work was performed to determine the conformational response in Env and potential disruption in the membrane to treatment with KR13 (and contrast with the thiol-blocked KR13b), by examining the epitopes of key Env regions and the permeability of the membrane to fluorescent dyes. By doing this, we aimed to learn the extent to which KR13-induced virolysis and transformation of the Env-membrane complex on cells resembles the native fusion transformation of Env. Env-presenting cells were used to facilitate analysis by flow cytometry, rather than examining residual virions, and the lack of CD4^+^/CoR^+^ target cells in the system ensured there would be no conflicting triggers of transformation.

As defined earlier in results, the bulk cell membrane differs from the final budded virus membranes [32], though Env can cluster and associate with cholesterol even in the absence of supporting viral proteins such as Gag [31,33], and cell-presented Env retains enough functionality for cell–cell fusion assays [34,35,36]. Moreover, the survival of Env-presenting cells through KR13 treatment, in contrast to the shriveled and disordered morphology of KR13-treated virions [23], may be in part due to the more heterogeneous composition of cell membranes compared to the more defined and rigid virus membrane.

### 4.2. Proposed Sinking Trimer Model for PTT-Induced Env Transformation and Exposure of Native-Fusion-Like Phenotypes

**Figure 6:**
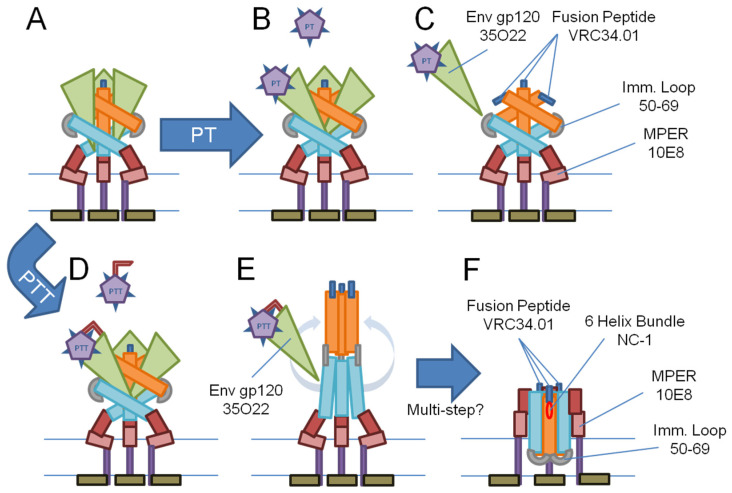
Proposed model describing the interaction of PT and PTT compounds with the HIV-1 Env trimer and the ensuing transformations of component protomers. (**A**) Ground state of membrane-embedded, fusion-competent HIV-1 Env. (**B**) The IXW pharmacophore of PT binds at a site straddling the CD4 and co-receptor binding sites of Env gp120 [27]. (**C**) PT causes gp120 shedding but does not trigger fusion-related transformations [23]. (**D**) PTT binds to gp120′s CD4/co-receptor sites as in B, but it engages in an additional thiol–Env exchange secondary to pharmacophore binding [26]. (**E**) PTT causes gp120 shedding and additionally triggers transformation into the pre-fusion intermediate [23]. Subsequent processes may occur in a multi-step fashion subject to conformational equilibrium, leading to the final 6-helix-bundle form in F. (**F**) CHR helices (cyan) fold up and into grooves of the NHR trimer (orange) to form the 6-helix-bundle, pushing the immunodominant loop end into the membrane, and dredging MPER up from the membrane; membrane disruption occurs.

Based on the results of this study, we constructed a model (Figure 6) to describe our observations of the PT- and PTT-induced rearrangements of Env. These rearrangements are envisioned in the context of the structural components, built into Env’s native metastable complex, that are known to be used for virus–cell fusion and entry [6,7,8].

Three key phenotypes observed after PTT treatment were MPER exposure (10E8 binding), 6-helix-bundle formation (NC-1 binding), and membrane disruption (calcein leakage). All three of these changes align with similar phenotypes expressed as a result of the native fusion process progressing through CD4 and co-receptor engagement and subsequent conformational rearrangement resulting in MPER exposure, 6-helix-bundle formation, and nondestructive opening of the fusion pore (analogous to PTT-induced membrane disruption) [5,10,18,43,55,56]. The reduced availability of the immunodominant loop (50-69) and fusion peptide (VRC34.01) epitopes in response to PTT treatment could also be signs of mechanisms expropriated from native fusion. Env-mediated cell–cell fusion experiments have found that areas of contact between Env and target cells bind cluster I (e.g., 50-69) or cluster II (e.g., 98-6) antibodies at up to 30 min after initial co-culture, but this disappears in syncytia and actively fusing cells, suggesting that the immunodominant loop region becomes unavailable during fusion [57]. Furthermore, the gp41 immunodominant loop region is hydrophobic and capable of both gp41 6-helix-bundle stabilization and membrane insertion itself during late-stage fusion [58,59,60,61]. Studies on HIV-1′s fusion peptide have reported that it is capable of interacting with both the MPER and transmembrane domain and promotes lipid mixing when bundled together [62,63]. Fusion peptide in pre-fusion Env trimers has also been shown to be conformationally flexible and solvent-exposed in “closed” state, but sequestered or buried in “open” and potentially intermediate pre-fusion states [64,65]. If a functional membrane-inserting fusion peptide is not involved with the PTT-induced transformation, as suggested by the V504E mutant results (Figure 4), the fusion peptide may retain sufficient flexibility to be sequestered within the protein complex and reduce its availability as observed after PTT exposure (Figure 3A), rather than having reduced availability from being membrane-buried.

The model of PTT-induced Env transformation and membrane disruption proposed in Figure 6 contrasts with the prototypical model of Env-driven fusion pore formation involving SNARE-like zippering of multiple 6-helix-bundles laterally around the fusion pore [10,66,67], as our observations in the current work disagree with features of that prototypical model. These include increased exposure of the immunodominant loop and fusion peptide epitopes during the prototypical fusion mechanism, while reduced exposure of these epitopes is observed for KR13-induced transformation (Figure 3A and Table 1). Furthermore, KR13 causes membrane leakage in the absence of an opposing membrane [22,23]. At this stage, while we believe that KR13-induced Env rearrangement and membrane disruption exploit conformational triggers intrinsic to Env in virus–cell fusion, future investigations will be needed to further correlate the PTT-induced Env inactivation and native fusion processes.

KR13b was found to trigger loss of gp120 (reduced 35O22 binding) at comparable concentrations to KR13 and with similar magnitude, which is in line with our existing understanding of PT and PTT mechanics. Shedding of gp120 is the primary influence on infection inhibition by both PT and PTT at relatively low concentrations [23]. The other transformations observed after KR13b treatment, increasing exposure of the immunodominant loop (50-69) and the fusion peptide (VRC34.01), suggest that these sites are natively occluded by the presence of gp120, or are otherwise stabilized by gp120′s removal. This also resembles some effects observed after treatment with soluble CD4, which also induces gp120 shedding, as well as increased exposure of cluster 1 antibodies (e.g., 50-69) and VRC34.01 [68].

### 4.3. Grouping of Epitope Responses into Low- and High-Dose Effects May Reflect Required Stoichiometries of Env/Ptt Interactions and Asymmetries in Trimer Transformation

The transitional effects observed in Figure 3 and Table 1 were found to have EC_50_ values that group around two dosage levels of KR13 and KR13b, approximately 0.3 µM and 1.0–1.5 µM. As the design of our experiments is more thermodynamics-oriented than kinetics-oriented, these groupings may indicate stoichiometric requirements and/or asymmetric interactions of Env/PT/PTT transformation more so than describing the exact sequence of mechanistic events. The low concentration (~0.3 µM) of KR13 or KR13b may be sufficient to trigger gp120 shedding by interacting with less than three protomers from a given Env trimer on average, whereas the higher concentration (~1.0 to 1.5 µM) may be needed to saturate three gp120 protomers of the Env trimer and elicit further responses translated to gp41.

For KR13b, gp120 shedding without associated thiol exchange (Figure 6B) may not significantly alter any other epitope presentation until the spike is fully “bare” of gp120 (i.e., Figure 3B at 0.625 µM, where gp120 is approaching a minimum and immunodominant loop and fusion peptide exposure are beginning to increase), at which point immunodominant loop and fusion peptide exposure would rise in fully exposed trimers (Figure 6C). In contrast, for the KR13 response, gp120 shedding plus thiol exchange (Figure 6D) could lead to immediate rearrangements to reduce immunodominant loop and fusion peptide exposure in the associated single gp41 protomer, whereas activating all three gp120 protomers plus thiol exchanges may be required to drive complete fusion-like rearrangement of pre-hairpin intermediate through to the final 6-helix-bundle conformation (Figure 6E,F). These differences may result from a conformational equilibrium of gp41 states elicited by more partial or more complete transformations induced by KR13 or KR13b, as each spike of trimeric Env offers three potential binding sites for KR13 or KR13b interaction. While native and untransformed Env trimers have been the subject of several studies examining open and closed ground states [68,69,70,71], less attention thus far has been focused on the state transformations from pre-hairpin intermediate through to the 6-helix-bundle formation; KR13 and PTT compounds, in general, may offer a method to reach that stage for further conformational experiments.

The data leading to our model only further emphasize the mechanistic difference the addition of the free thiol makes for PTT compounds, as well as the need to further investigate the cascade of interactions past the thiol/Env C296-C331 disulfide exchange. For similar reasons, kinetic experiments for the exposure of individual epitopes could also contribute to establishing a clear order of events after PTT/Env engagement. Asymmetric and mixed trimers on cell surfaces would also provide a valuable avenue of investigation and would extend prior mixed trimer studies performed in pseudovirus [72]. Previously, the S375W mutation was used to confer PT-pharmacophore binding resistance in pseudovirus produced from varying ratios of WT:S375W transfection. While proportions of up to 50% S375W DNA caused mild increases in virolysis EC_50_, 75% S375W DNA and higher also reduced the degree of lysis observed, fully negating it at 100% S375W DNA. Assuming independent assortment of WT and S375W protomers, the study concluded that KR13-induced virolysis would need a statistical average of at least one fully active Env spike (three WT protomers in the trimer) to be present on the virus surface. Extending this to describe the conformation and epitope presentation of such mixed trimers could be a very fruitful follow-up study to learn more about the roles of stoichiometry and asymmetric occupancy in Env transformation triggered by PT and PTT.

### 4.4. Differential Inhibition of PTT-Induced MPER Exposure and Membrane Disruption by 6-Helix-Bundle Inhibitor T20 Suggests More Complex Transformations

The T20 experiment (Figure 5) included in this study was our initial attempt to clarify the order of conformational rearrangements on Env, but ultimately showed that the transformation may be more complex than initially suspected. T20 was selected as an inhibitor of 6-helix-bundle formation, a deliberate backstop to halt transformation at a known stage. However, this time, we saw separate dose-dependent effects for T20 inhibition of KR13-induced phenotypes of membrane disruption and MPER exposure. Shedding of gp120 was unaffected by T20 concentration, which is in line with the understanding of shedding as an early event occurring upstream of 6-helix-bundle formation. Meanwhile, low doses of T20 (0.19±0.03 µM) were required to inhibit MPER/10E8 exposure, and high doses (2.26±0.24 µM) to inhibit membrane disruption/calcein leakage. Fitting this into the proposed model in Figure 6 may also suggest a more complex conformational equilibrium between pre-hairpin intermediate and the 6-helix-bundle hairpin structure, as the high sensitivity of MPER exposure and low sensitivity of membrane disruption to inhibition argues against 6-helix-bundle formation and membrane disruption as a single concerted event. Further studies, especially targeted at the kinetics of T20 inhibition of PTT-induced transformations, will be necessary to form more definitive conclusions. 

### 4.5. Incomplete Conversion of Env Observed on PT and PTT Treated Cell Surfaces

As shown in Figure 3, even treatment with relatively high concentrations of KR13/KR13b did not result in complete Env transformation on all cells. This may have resulted from the 4-h time exposure. Longer treatment times can result in more complete transformation, analogous to previous pseudovirus studies [23]. However, longer treatment times were avoided in the current cell-based study, since they may also lead to the complicating factor of new Env production and transport to the cell membrane, or recycling of transformed Env from the membrane. This could be countered in future studies by additional use of transport and retrograde inhibitors, such as Brefeldin A or *Legionella*’s RidL [73,74]. Disulfide exchange with gp120 may also be a concern, particularly for the KR13 results. As previously established, KR13′s free thiol likely initiates a disulfide exchange cascade [26], but in doing so, it forms a covalent mixed disulfide bond between its thiol and that of the first disulfide (likely C296-C331), removing itself as a potential agent against subsequent Env proteins. Given that the high end of treatment has several data points that plateau, exhausting KR13 is likely not the entire explanation for incomplete conversion. Variation in cell transfection and Env expression may play an important role: while almost all cells showed some level of transfection (efficiency ~90 to 95%), there were bidisperse populations observed for all of the epitopes, showing higher and lower levels of Env expression, possibly caused by uneven transfection with the JRFL Env-encoding plasmid. Another potential explanation may be a population of defective spikes produced on the cell surface, exacerbated by the HEK293T cell line’s relative ease of transfection and production of viral proteins. “Decoy” defective Env forms have been observed on cells and viruses, including uncleaved gp160 and “stump” gp41, with the purpose of aiding immune evasion due to promoting non-neutralizing responses or misdirecting neutralizing responses toward non-functional viruses or cells [75,76,77]. Despite these incomplete conversions, clear dose-dependent changes in epitope exposure were observed in response to PT and PTT treatment, indicating that conformational rearrangements were induced in Env.

### 4.6. Transient Membrane Disruption Implies Temporary and Size-Limited Poration by PTT

We did not observe appreciable loss of cells or cell viability from KR13 or KR13b treatment, in direct contrast to another entry inhibitor that we tested against Env-presenting cells in a previous study, a microvirin-MPER based chimeric protein [37]. This would imply that membrane disruption/poration induced by KR13 is transient and limited in size, such that it does not result in cell death and thus must be repairable/survivable. This controlled pore formation may be another similarity to native fusion, in disrupting the membrane without causing permanent and fatal damage to the cell, analogous to forming the fusion pore through which viral RNA and reverse transcriptase are delivered. This is also in contrast to the KR13-induced lysis of virus, which, lacking the cell’s machinery and capacity for membrane repair, appears to have no recourse but to continue leaking and deflate [23]. Furthermore, the intracellular dye used here in the cellular studies was calcein, which has a hydrodynamic radius of approximately 1.3 nm [78]. This ensures that it may diffuse out through almost any size membrane disruption (including the transient effects observed in this study), but it tells us less about how large a given disruption may be. For future studies on membrane disruption and pore formation by Env/PTT, bulkier dyes, such as polydextran linked fluorophores, could provide larger markers to determine the size of individual disruptions, as in pore-forming toxin studies [79,80].

### 4.7. Impact and Future Directions

In this study, we demonstrated that the peptide triazole thiol KR13 causes nonlethal membrane disruption and conformational change in cell-presented Env. We also specifically contrasted these results with those from KR13b treatment, a peptide identical to KR13, save for its disabled thiol, bringing further evidence that it is the free thiol functionality of KR13 that results in the membrane disruption and Env rearrangement phenotypes. The T20 inhibition of KR13 experiment further indicated that the KR13-triggered membrane disruption and MPER exposure were both dependent on the ability of the Env to form the 6-helix-bundle, suggesting that, for KR13 treatment, MPER exposure is an end-state, rather than a transiently available epitope, but may also rely on a multi-step process of rearrangement.

The combined results obtained in the current study invite further evaluation of KR13 and other PTT compounds on membrane-embedded Env trimers (cells, viruses, and lipid nanodisc platforms) as a method to induce conformational phenotypes for immunogen development, particularly targeting MPER and other sites enabling broad neutralization. Mechanistically, we will continue investigation of the conformational equilibrium between pre-hairpin and hairpin structures by kinetic and structure-based experiments to complement the more thermodynamic perspective in this study. In particular, mixed hetero-trimer experiments incorporating PT-resistant Env may be invaluable for disentangling potential states and transformations between pre-hairpin and hairpin structures as described by epitope mapping. Additionally, the confirmation of the free thiol being key to membrane disruption and Env transformation will allow us to further test mechanistic similarities between KR13-induced transformation and native fusion, by specifically examining critical thiol/disulfide interactions during the native fusion process and testing if they are relevant to KR13 transformations [26,81,82,83].

## Figures and Tables

**Table 1:** Collected EC_50_ values for KR13 and KR13b transformations with relative change in exposure. EC_50_ values are presented as *x*_0_ ± the standard error of *x*_0_.

Antibody/Epitope	KR13	KR13b
Calcein Membrane Disruption	1.15 ± 0.02 µM Decrease	No Change
35O22 gp120 Shedding	0.33 ± 0.02 µM Decrease	0.28 ± 0.05 µM Decrease
10E8 MPER	1.42 ± 0.32 µM Increase	No Change
NC-1 6-Helix-Bundle	1.49 ± 0.26 µM Increase	No Change
50-69 Immunodominant Loop	0.24 ± 0.05 µM Decrease	1.02 ± 0.13 µM Increase
VRC34.01 Fusion Peptide	0.28 ± 0.06 µM Decrease	1.26 ± 0.10 µM Increase

## Data Availability

The analyzed data presented in this study are included within this article and supplemental materials. Further data is available on reasonable request from the corresponding authors.

## Supplementary Materials

The following are available online at https://www.mdpi.com/article/10.3390/microorganisms9061286/s1, Figure S1: Graphs for dye retention and epitope exposure response to KR13 and KR13b, separated by dye/epitope, Figure S2: Lack of cytotoxicity in JRFL Env expressing and non-expressing HEK293T cells after 24 h treatment with KR13 or KR13b.
