# Supplementary material for: Peptide Triazole Thiol Irreversibly Inactivates Metastable HIV-1 Env by Accessing Conformational Triggers Intrinsic to Virus–Cell Entry
Source: Microorganisms. 2021 Jun 12;9(6):1286. doi: 10.3390/microorganisms9061286 (PMC8231586; doi:10.3390/microorganisms9061286)
Supplement: Supplementary file 1 [file microorganisms-09-01286-s001.zip › microorganisms-1211897-supplementary.pdf]

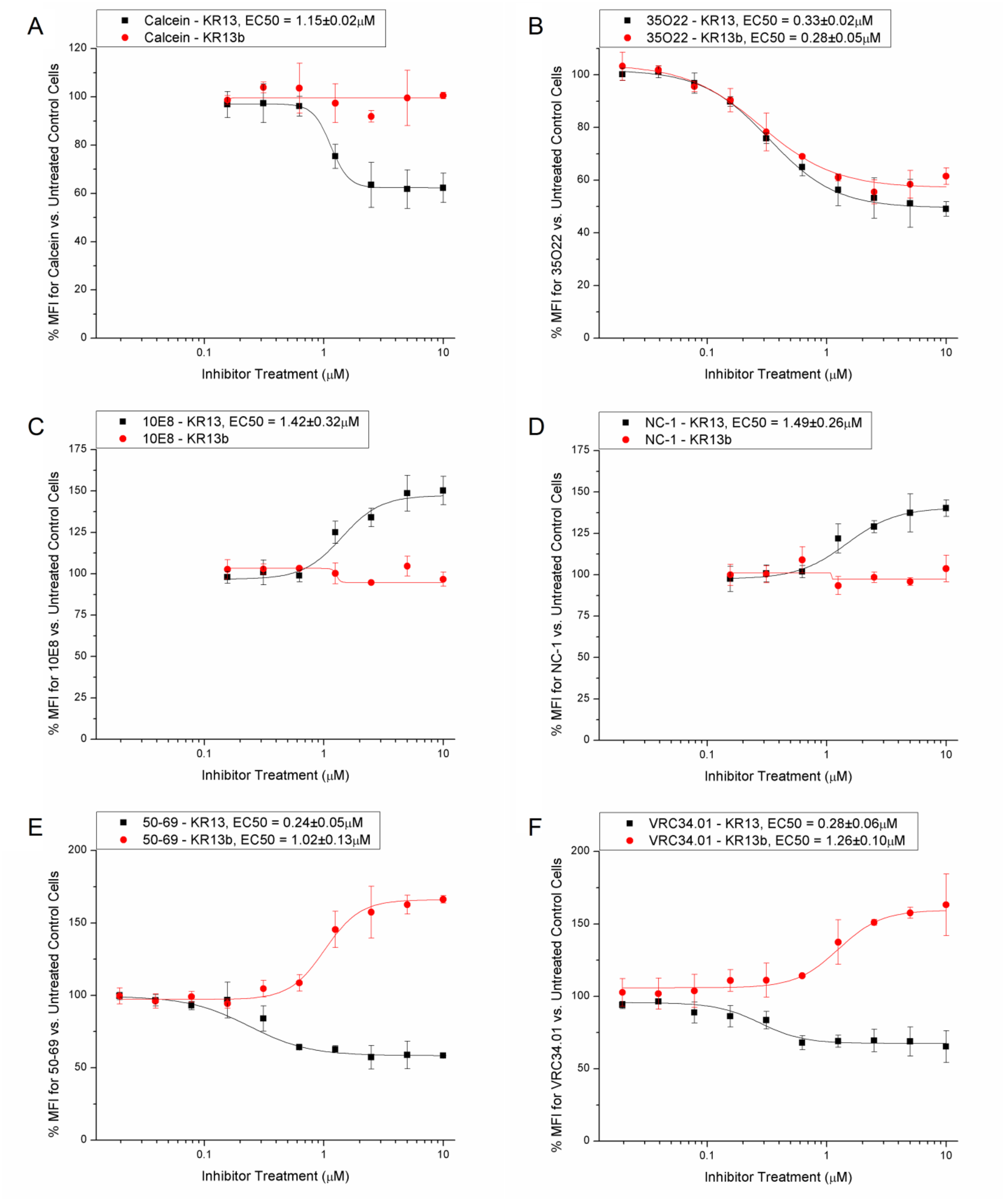


**Figure S1:** Graphs for dye retention and epitope exposure response to KR13 and KR13b, separated by dye/epitope. (**A**) Calcein dye retention, membrane integrity. (**B**) Ab 35O22, gp120/gp41 interface. (**C**) Ab 10E8, MPER. (**D**) Ab NC-1, 6-helix bundle. (**E**) 50–69, immunodominant loop. (**F**) VRC34.01, fusion peptide.

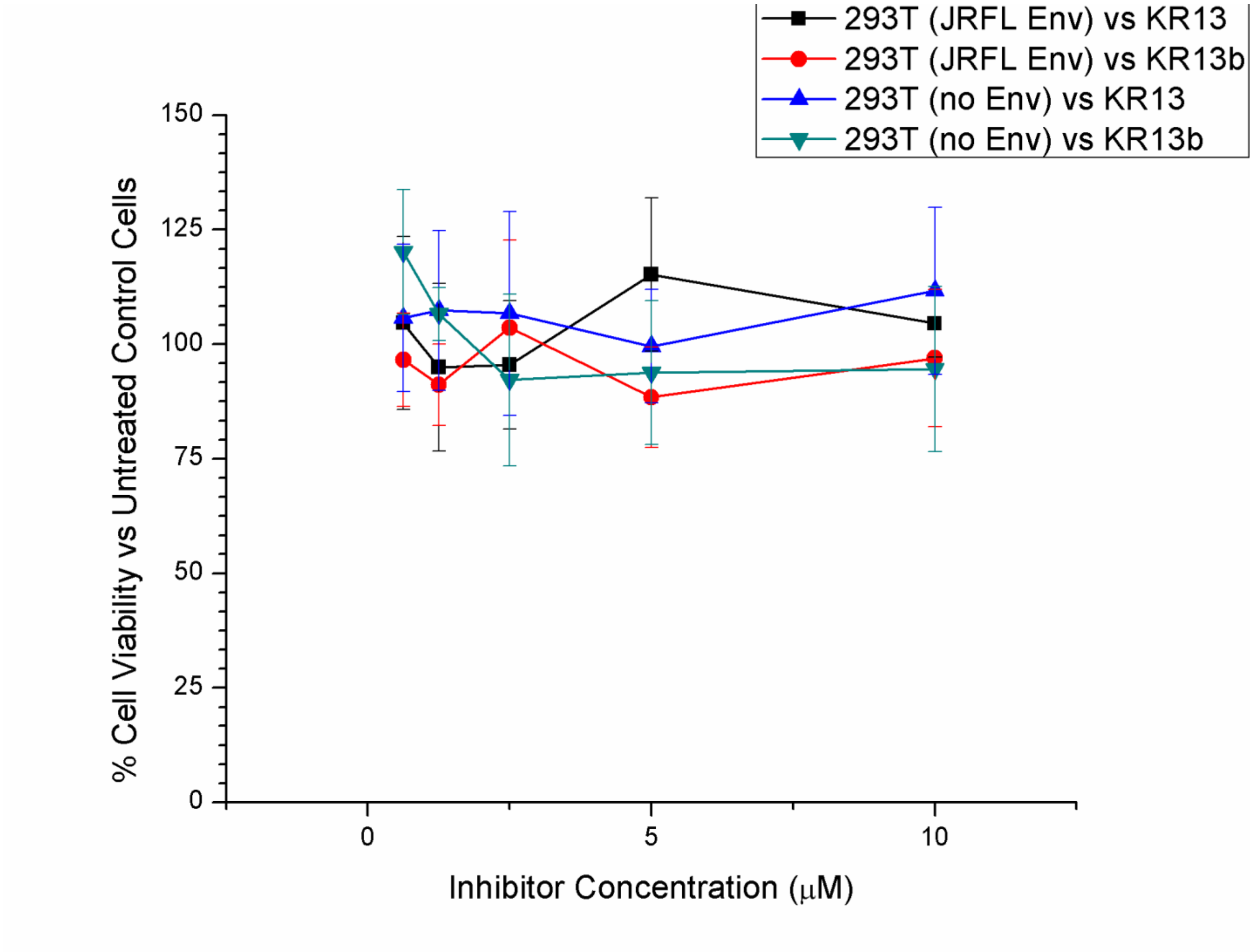


**Figure S2:** Lack of cytotoxicity in JRFL Env expressing and non-expressing HEK293T cells after 24 h treatment with KR13 or KR13b. Transfected and untransfected 293T cells were incubated for 24 h at 37 °C with serial dilutions of KR13, KR13b, or PBS control. Cells were then washed with media, then incubated with 10% WST-1 Cell Proliferation Reagent (Takara Bio USA, Inc.; Mountain View, CA) in media for 1h, before measurement of absorbance at 450 nm using a Tecan Infinite F50 plate reader (Männedorf, Switzerland), indicative of mitochondrial activity and cell viability. Data shown are the average of three independent experiments and error bars show the standard deviation of the mean.
